# Revisiting interaction specificity reveals neuronal and adipocyte Munc18 membrane fusion regulatory proteins differ in their binding interactions with partner SNARE Syntaxins

**DOI:** 10.1371/journal.pone.0187302

**Published:** 2017-10-31

**Authors:** Michelle P. Christie, Shu-Hong Hu, Andrew E. Whitten, Asma Rehman, Russell J. Jarrott, Gordon J. King, Brett M. Collins, Jennifer L. Martin

**Affiliations:** 1 Division of Chemistry and Structural Biology, Institute for Molecular Bioscience, University of Queensland, St Lucia, Queensland, Australia; 2 Division of Cell Biology and Molecular Medicine, Institute for Molecular Bioscience, University of Queensland, St Lucia, Queensland, Australia; 3 Griffith Institute for Drug Discovery, Griffith University, Nathan, Queensland, Australia; University of Cincinnati College of Medicine, UNITED STATES

## Abstract

The efficient delivery of cellular cargo relies on the fusion of cargo-carrying vesicles with the correct membrane at the correct time. These spatiotemporal fusion events occur when SNARE proteins on the vesicle interact with cognate SNARE proteins on the target membrane. Regulatory Munc18 proteins are thought to contribute to SNARE interaction specificity through interaction with the SNARE protein Syntaxin. Neuronal Munc18a interacts with Syntaxin1 but not Syntaxin4, and adipocyte Munc18c interacts with Syntaxin4 but not Syntaxin1. Here we show that this accepted view of specificity needs revision. We find that Munc18c interacts with both Syntaxin4 and Syntaxin1, and appears to bind “non-cognate” Syntaxin1 a little more tightly than Syntaxin4. Munc18a binds Syntaxin1 and Syntaxin4, though it interacts with its cognate Syntaxin1 much more tightly. We also observed that when bound to non-cognate Munc18c, Syntaxin1 captures its neuronal SNARE partners SNAP25 and VAMP2, and Munc18c can bind to pre-formed neuronal SNARE ternary complex. These findings reveal that Munc18a and Munc18c bind Syntaxins differently. Munc18c relies principally on the Syntaxin N-peptide interaction for binding Syntaxin4 or Syntaxin1, whereas Munc18a can bind Syntaxin1 tightly whether or not the Syntaxin1 N-peptide is present. We conclude that Munc18a and Munc18c differ in their binding interactions with Syntaxins: Munc18a has two tight binding modes/sites for Syntaxins as defined previously but Munc18c has just one that requires the N-peptide. These results indicate that the interactions between Munc18 and Syntaxin proteins, and the consequences for *in vivo* function, are more complex than can be accounted for by binding specificity alone.

## Introduction

Soluble N-ethylmaleimide sensitive factor attachment protein receptor (SNARE) proteins mediate vesicle docking and fusion involved in the transport of cellular cargo. Assembly of this SNARE complex brings the fusing membranes together and provides the energy required to drive membrane fusion. At the heart of the SNARE hypothesis it is proposed that specific SNARE partner combinations are involved in different transport events. For example, the SNARE proteins Syntaxin4 (Sx4), SNAP23 and VAMP2 are required for the fusion of glucose transporter GLUT4 storage vesicles (GSVs) with the plasma membrane in muscle and adipose tissues. In contrast, neurons utilise the related but distinct t-SNAREs Syntaxin1 (Sx1) and SNAP25 that pair with the v-SNARE VAMP2 on synaptic vesicles to control neurotransmitter release required for neurotransmission.

SNARE mediated fusion is further regulated by essential Sec1/Munc18 (SM) family proteins. SM proteins are SNARE binding proteins that play a crucial role in the late stages of vesicle docking and fusion, as well as stabilisation of the target Syntaxin (Sx) proteins. The SM proteins are a highly conserved protein family that function at membrane interfaces throughout the cell [1]. In mammals, three SM proteins—Munc18a, Munc18b and Munc18c –regulate transport to the plasma membrane. Munc18a (n-Sec1, Munc18-1) was identified as a Sx1-binding protein in brain lysates [2]. Munc18b (Munc18-2) and Munc18c (Munc18-3) isoforms were subsequently identified [3]. Munc18a is expressed predominantly in the brain, whereas Munc18b and Munc18c appear to be ubiquitously expressed.

Munc18 loss-of-function or null mutations abrogate or severely impair fusion [4–7]. However the precise role these proteins play in fusion remains contentious; with both positive and negative functions reported. This is in part due to different binding modes that Munc18 displays with its cognate Sx. One binding mode involves the binding of multiple Sx domains to the Munc18 protein and appears to be consistent with a closed or non-fusion competent state of the Sx [8, 9]. Another binding mode, observed between Sx4 and Munc18c, requires just the N-terminal ten residues (N-peptide) of the Sx [10, 11]. This second binding mode is consistent with an open Sx4 conformation, suggesting a positive role for Munc18c. Indeed, the Munc18:N-peptide interaction has been shown to be universally important for interaction of Munc18s with SNARE complexes [12, 13] with a positive regulatory role implicated for Munc18a [13, 14].

Munc18:Sx interactions are thought to contribute to membrane fusion specificity [13, 15–17]. In this paradigm, Munc18a binds to Sx1 but not Sx4, whereas Munc18c binds to Sx4 but not Sx1 [17, 18]. However, these Munc18:Sx partnerships were identified in large part from early *in vitro* work, and before the importance of the N-peptide was appreciated. Munc18:Sx specificity has not been revisited since then. It is noteworthy that the Sx1/Sx4 N-peptide sequences are almost identical and both N-peptides bind to Munc18a (*K*_d_ Sx1 N-peptide ~60 μM; Sx4 N-peptide ~30 μM) and to Munc18c (*K*_d_ Sx4 N-peptide ~2 μM; Sx1 N-peptide ~20 μM) [19]. This suggests two possibilities—either there is no specificity for the Munc18:Sx interaction between these pairs of proteins or the specificity is defined by interactions other than the N-peptide binding site.

The present work revisits the specificity of interactions between Munc18 and Sx proteins, using *in vitro* binding experiments focusing on Munc18a and Munc18c and soluble truncated forms of Sx4 and Sx1. We found, contrary to previous reports, that Munc18c interacts equally well with both Sx4 and Sx1, and that Munc18a interacts with Sx4, though not as tightly as it interacts with its cognate Sx1. Notably, the Sx N-peptide is critical for tight-binding of Sx1 and Sx4 with Munc18c, and for the interaction between Sx4 and Munc18a, but is not so critical for the tight interaction between Sx1 and Munc18a.

## Results

### Munc18c and Munc18a bind “non-cognate” Sx partners

**Fig 1:**
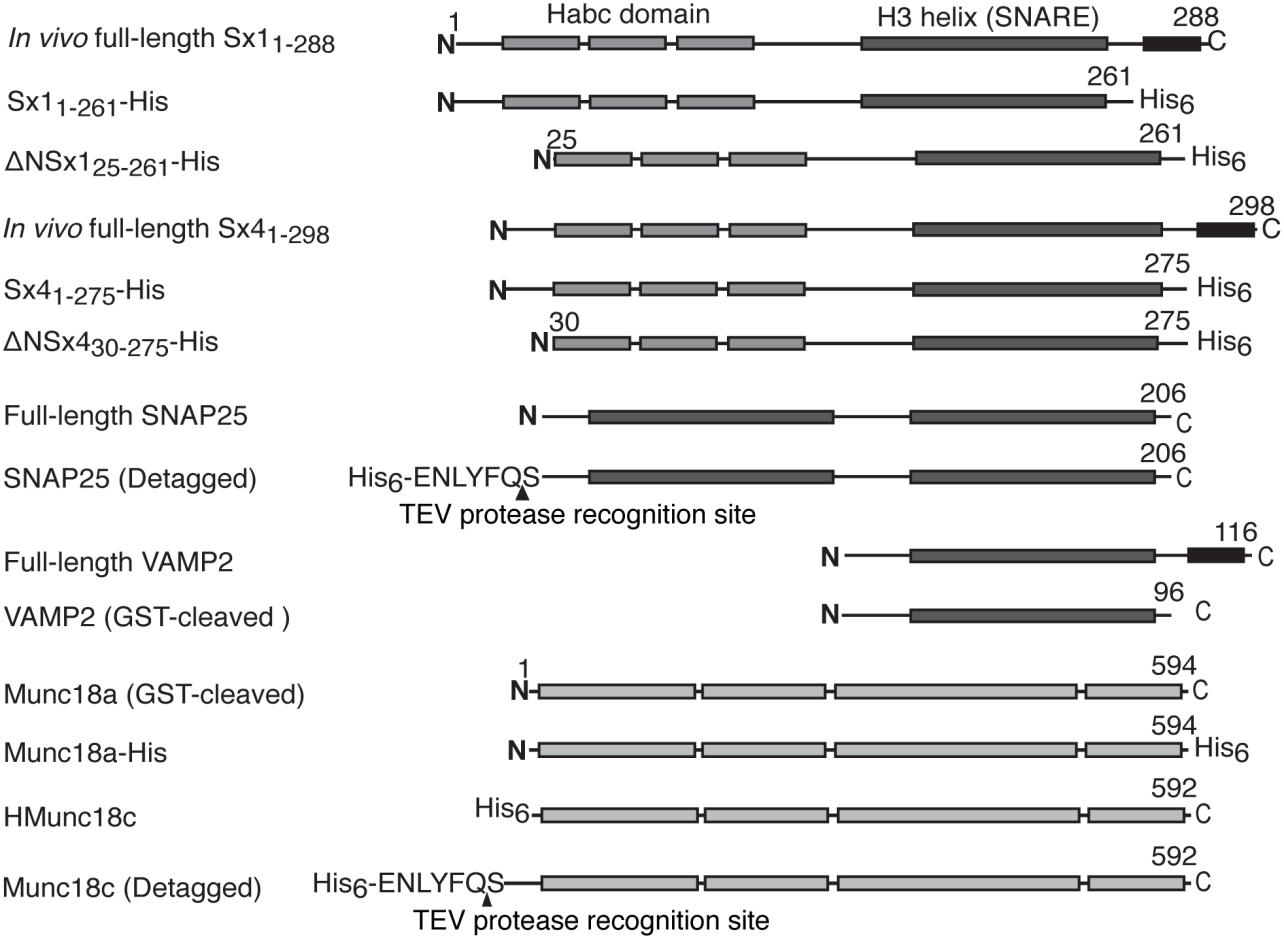
Schematic representation of the protein constructs used in this study. *In vivo* full-length Sx consists of an N-peptide preceding an N-terminal α-helical bundle (the H_abc_ domain), a SNARE motif (the H3 helix) and a C-terminal transmembrane region. We used soluble Sx1 and Sx4 constructs lacking the transmembrane domain for experiments reported here. ΔN indicates Sx constructs lacking the N-peptide. Munc18 and SNAP25 and VAMP2 constructs used in these experiments are also shown. The positions of engineered fusion tags and protease cleavage sites are as indicated.

According to the current paradigm, the interaction between Munc18 and Sx is specific such that Munc18a binds Sx1 but not Sx4, while the homologous protein Munc18c binds Sx4 but not Sx1. To determine if this was indeed the case, we used soluble Sx1 and Sx4 constructs (Fig 1) in which the C-terminal Sx transmembrane domains were replaced with C-terminal His_6_ tags for *in vitro* binding experiments with Munc18a and Munc18c. We used Sxs engineered with a C-terminal His_6_ tag, rather than N-terminal affinity tags used in previously reported *in vitro* experiments [17, 18, 20], because this arrangement ensured that the Sx N-peptides were available to interact with Munc18 proteins.

**Fig 2:**
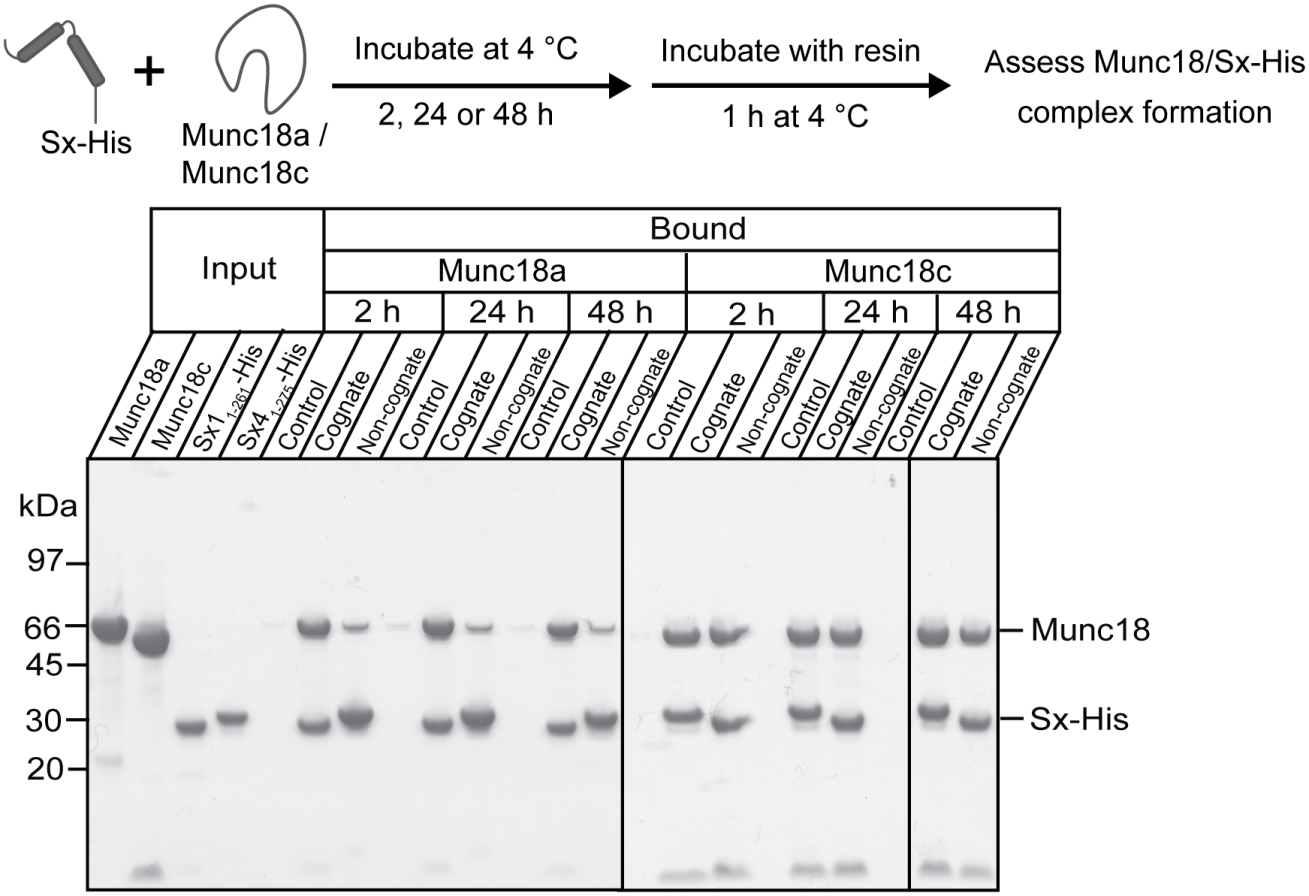
Munc18 proteins bind non-cognate Sxs. Sx1_1-261_-His or Sx4_1-275_-His were incubated with detagged Munc18a or Munc18c for 2 h, 24 h or 48 h before the Sx was immobilized onto TALON^™^ Co^2+^ affinity beads for 1h and then washed. Samples of the beads were then run on SDS-PAGE and stained with Coomassie Blue to determine if detagged Munc18 had been pulled down by cognate and non-cognate Sx partners. Detagged Munc18a and Munc18c were also incubated for the same time periods on beads without bound Sx to monitor non-specific binding (control lanes). Solid vertical lines on the gel image denote the removal of intervening lanes or placing two different gels adjacent to each other.

In control experiments, Sx proteins captured similar amounts of their cognate partners (Munc18a:Sx1_1-261_-His; Munc18c:Sx4_1-275_-His, Fig 2). However, in contradiction to the current paradigm, we found that both Sxs captured “non-cognate” Munc18s (Fig 2). Specifically, detagged Munc18a was captured by Sx4_1-275_-His, albeit more weakly than by Sx1_1-261_-His (Fig 2). More surprising, Sx1_1-261_-His and Sx4_1-275_-His captured similar amounts of Munc18c (detagged) (Fig 2). Taken together, these results suggest that Sx1 binds tightly to both Munc18a and Munc18c, whereas Sx4 binds tightly to Munc18c and more weakly to Munc18a. Alternatively, one can consider that Munc18c interacts tightly with both Sxs, and that Munc18a interacts more tightly with Sx1 and more weakly with Sx4.

**Fig 3:**
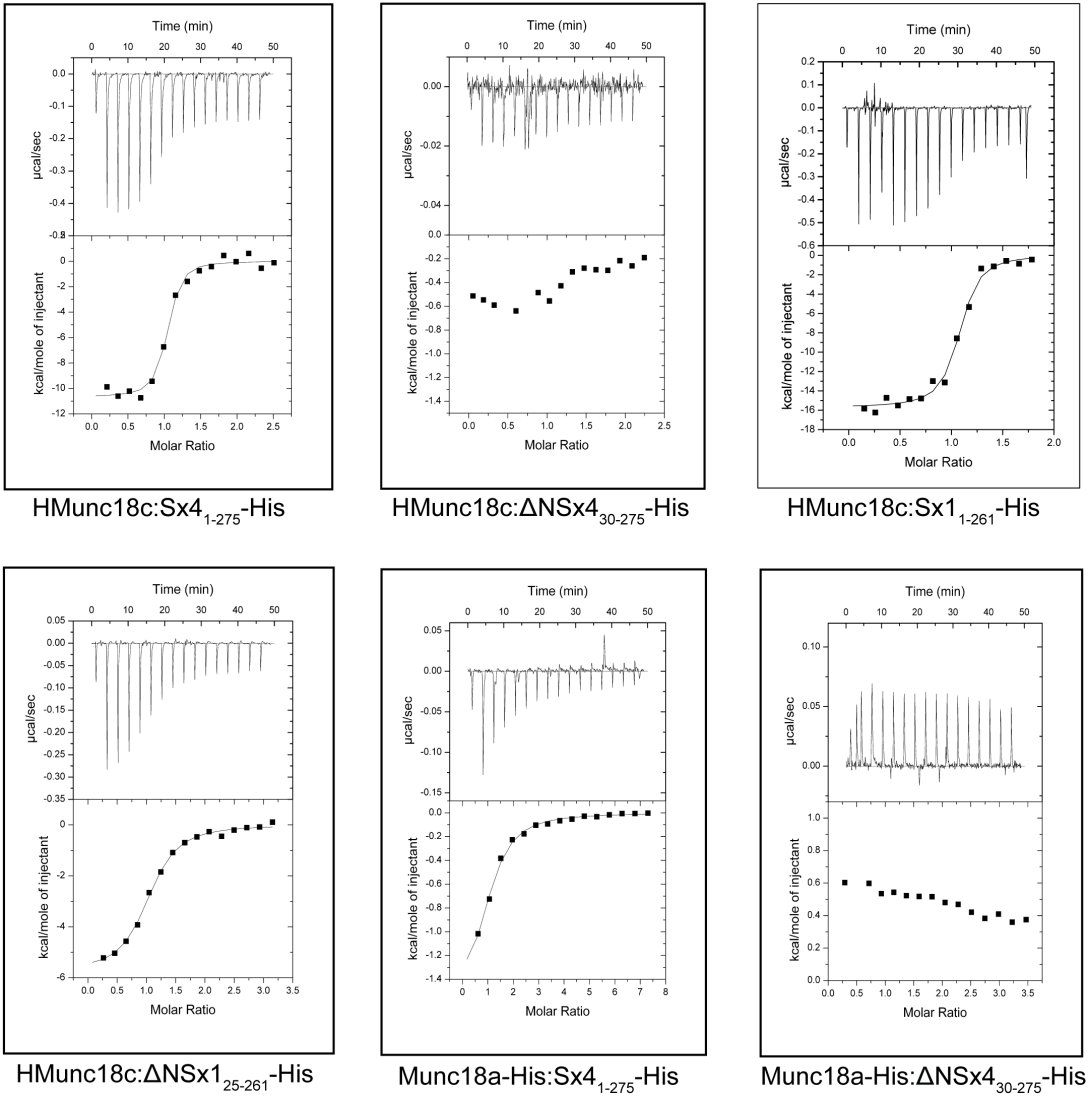
Isothermal titration calorimetry data. The raw data (upper part of each panel) and integrated normalized data (lower part of each panel) are shown from ITC experiments between HMunc18c or Munc18a-His and cognate/non-cognate Sx partners.

To quantify the binding affinity and thermodynamics of the interaction between these two Munc18 and two Sx proteins, we used isothermal titration calorimetry (ITC) (Table 1, Fig 3). Again we used truncated Sxs lacking their transmembrane domains for these measurements. ITC data reported by us and others previously have shown that under similar conditions Munc18a binds Sx1 (*K*_d_, ~1 nM) about 100-fold more tightly than Munc18c binds Sx4 (*K*_d_, ~100 nM) [21]. In the present work, we found a weak but detectable interaction between the non-cognate pair of proteins Munc18a-His and Sx4_1-275_-His by ITC (*K*_d_, 32 μM, Table 1). This affinity is ~300-fold weaker than the cognate Munc18c:Sx4_1-275_-His interaction and 30,000-fold weaker than that reported for the cognate Munc18a:Sx1_1-261_-His interaction (Table 1).

**Table 1:** ITC derived thermodynamic parameters for Munc18:Sx-His and Munc18:ΔNSx-His interactions. Values reported are average and standard deviation from at least three experiments.

Sx	Munc18	Cognate/ Non-cognate	N	Δ*H* (kcal/mol)	-Δ*G* (kcal/mol)	*K*_d_ (μM)
Sx4_1-275_-His	HMunc18c	Cognate	1.03 ± 0.02	-14.1 ± 0.6	4.5 ± 0.8	**0.10** ± **0.03**
N-pep_Sx4_	Munc18c*	Cognate	0.99 ± 0.02	-6.1 ± 1.0	-7.97 ± 0.07	**1.5** ± **0.2** [19]
ΔNSx4_30-275_-His	HMunc18c	Cognate	–	–	–	**–**
Sx4_1-275_-His	Munc18a-His	Non-cognate	1.02 ± 0.13	-3.1 ± 1.5	-3.1 ± 1.8	**32 ± 16**
N-pep_Sx4_	Munc18a-His	Non-cognate	1.10 ± 0.17	-2.6 ± 0.6	-6.15 ±0.12	**31 ± 8 [19]**
ΔNSx4_30-275_-His	Munc18a-His	Non-cognate	–	–	–	**–**
Sx1_1-261_-His	Munc18a-His	Cognate	-0.98 ± 0.02	-20.6 ± 0.9	-12.1 ± 0.1	~**0.001** [21]
N-pep_Sx1_	Munc18a-His	Cognate	1.0 ± 0.5	-3.8 ± 0.8	-5.8 ± 0.20	**60 ± 21 [19]**
ΔNSx1_25-261_-His	Munc18a-His	Cognate	1.05 ± 0.09	-12.0 ± 0.4	-11.0 ± 0.2	~**0.010** [21]
Sx1_1-261_-His	HMunc18c	Non-cognate	1.03 ± 0.01	-15.2 ± 0.6	5.5 ± 0.7	**0.08** ± **0.02**
N-pep_Sx1_	Munc18c*	Non-cognate	1.09 ± 0.09	-4.3 ± 0.4	-6.50 ± 0.25	**18 ± 7 [19]**
ΔNSx1_25-261_-His	HMunc18c	Non-cognate	1.07 ± 0.05	-4.44 ± 1.1	-3.9 ± 1.2	**730 ± 160**

Shading highlights the cognate and non-cognate interactions of Munc18c

*detagged Munc18c (recombinant expression from insect cells) [19]

We compared the ITC-determined binding affinities of HMunc18c for its cognate and non-cognate partners, Sx4_1-275_-His and Sx1_1-261_-His, respectively. We found that Munc18c bound “non-cognate” Sx1_1-261_-His very tightly (*K*_d_, 80 nM) with an affinity similar—even a little tighter—to that of “cognate” Sx4_1-275_-His (*K*_d_, 100 nM) (Table 1). Nevertheless, this HMunc18c:Sx1_1-261_-His interaction was ~80-fold weaker than the cognate Munc18a:Sx1_1-261_-His interaction (*K*_d_, 1 nM) largely due to a less favourable enthalpy [21, 22].

### The Sx4 interaction with Munc18c or Munc18a relies largely on its N-peptide

**Fig 4:**
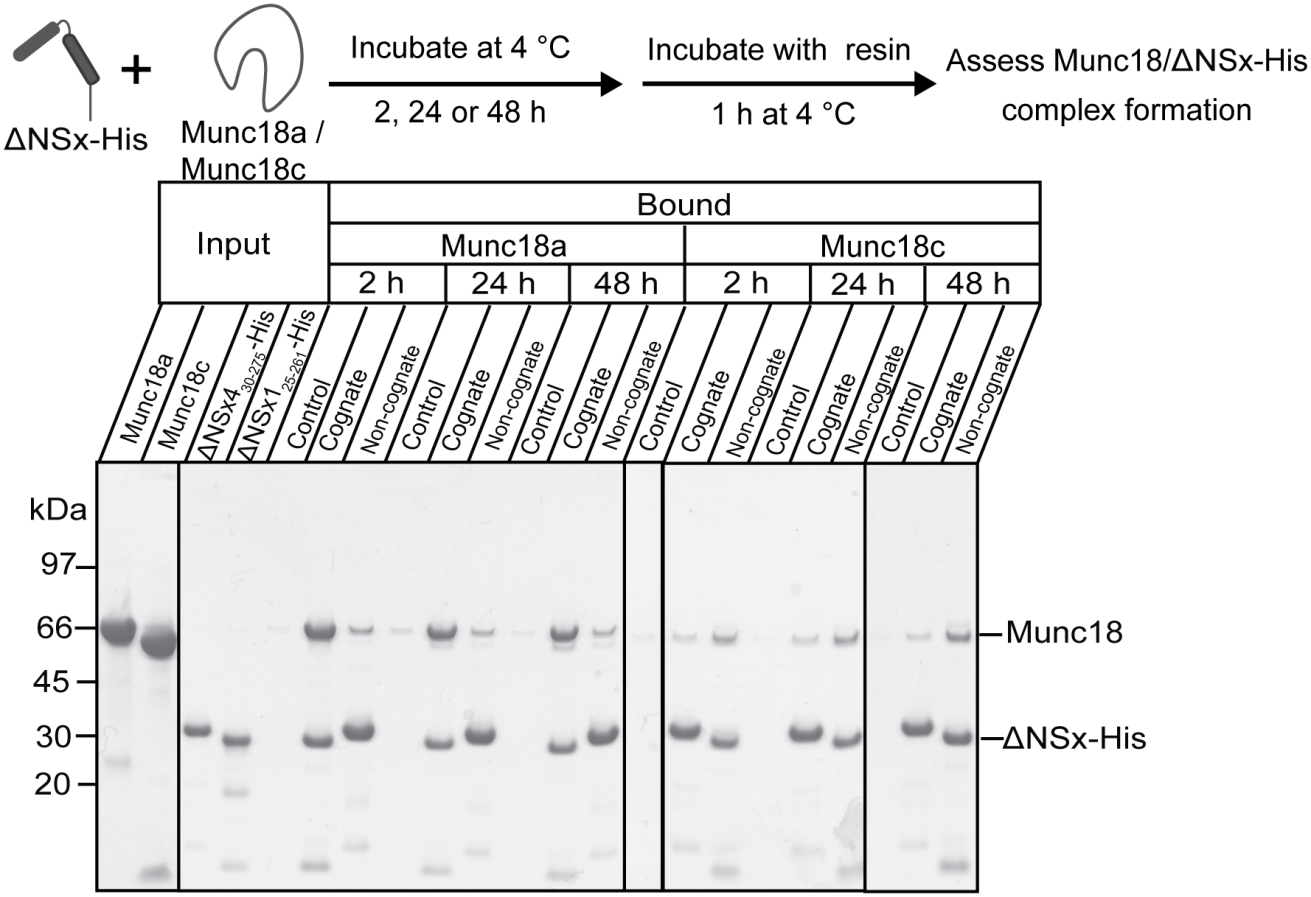
Munc18 proteins bind weakly to non-cognate ΔNSxs. ΔNSx1_25-261_-His or ΔNSx4_30-275_-His were incubated with detagged Munc18a or Munc18c for 2h, 24 h or 48 h before ΔNSx was immobilized onto TALON^™^ Co^2+^ affinity beads for 1h and then washed. Samples of the beads were then run on SDS-PAGE and stained with Coomassie Blue to determine if Munc18 proteins bound to ΔNSxs. Munc18 proteins were also incubated for the same time periods on beads without bound Sx to monitor non-specific binding (control lanes). Solid vertical lines on the gel image denote the removal of intervening lanes or placing two different gels adjacent to each other.

The Sx4 N-peptide is critical for its interaction with Munc18c, as evidenced by a lack of detectable binding between ΔNSx4 and Munc18c by ITC or by pulldowns [21]. These results were reproduced here; no binding was detected between ΔNSx4_30-275_-His and HMunc18c by ITC (Table 1) and detagged Munc18c was barely detectable in pulldowns with ΔNSx4_30-275_-His even after 48 h (Fig 4). Similarly, when we used the non-cognate partner Munc18a-His we were unable to detect an interaction by ITC with ΔNSx4_30-275_-His under the same conditions (Table 1) although ΔNSx4_30-275_-His captured some Munc18a in pulldown experiments (Fig 4). The ITC-determined binding affinity and thermodynamics for the non-cognate interaction between Munc18a and Sx4_1-275_-His are similar to the values we reported previously for the Munc18a interaction with the Sx4 N-peptide alone (Table 1). These results suggest that—whether it binds to Munc18c or to Munc18a –Sx4 relies to a large extent on its N-peptide interaction.

### Munc18a and Munc18c binding modes for Sxs differ

Previous reports of ITC-determined affinities show that Sx1_1-261_-His constructs with and without the N-peptide (ΔNSx1_25-261_-His) bind almost equally well to “cognate” Munc18a (*K*_d_, ~1 nM and 10 nM, respectively) [21, 22]. We showed above that Sx1_1-261_-His binds tightly to “non-cognate” HMunc18c. If the binding mode between Munc18c and Sx1_1-261_-His is the same as the binding mode between Munc18a and Sx1_1-261_-His, we would expect a similar pattern–*ie* ΔNSx1_25-261_-His binding affinity for Munc18c would be around 10-fold weaker than that of Sx1_1-261_-His. However, the interaction between HMunc18c and ΔNSx1_25-261_-His was four orders of magnitude weaker than for Sx1_1-261_-His (*K*_d_, 730 μM compared with 0.08 μM) and five orders of magnitude weaker than the interaction between ΔNSx1_25-261_-His and “cognate” Munc18a-His (*K*_d_, 0.01 μM) [21]. No interaction was detected between HMunc18c and ΔNSx4_30-275_-His using ITC.

We also performed pulldown experiments with the N-terminally truncated Sx-His proteins. These experiments confirmed the ITC data showing that Munc18a binds tightly to ΔNSx1_25-261_-His, and much more weakly to non-cognate ΔNSx4_30-275_-His and that Munc18c binds ΔNSx1_25-261_-His weakly and ΔNSx4_30-275_-His even more weakly (Fig 4).

Together these results support the notion that Munc18c relies to a large extent on the N-peptide interaction when it binds either Sx4 or Sx1. This is not the case for Munc18a and its interaction with Sx1.

### Sx1 bound to non-cognate Munc18c captures neuronal SNARE partners

**Fig 5:**
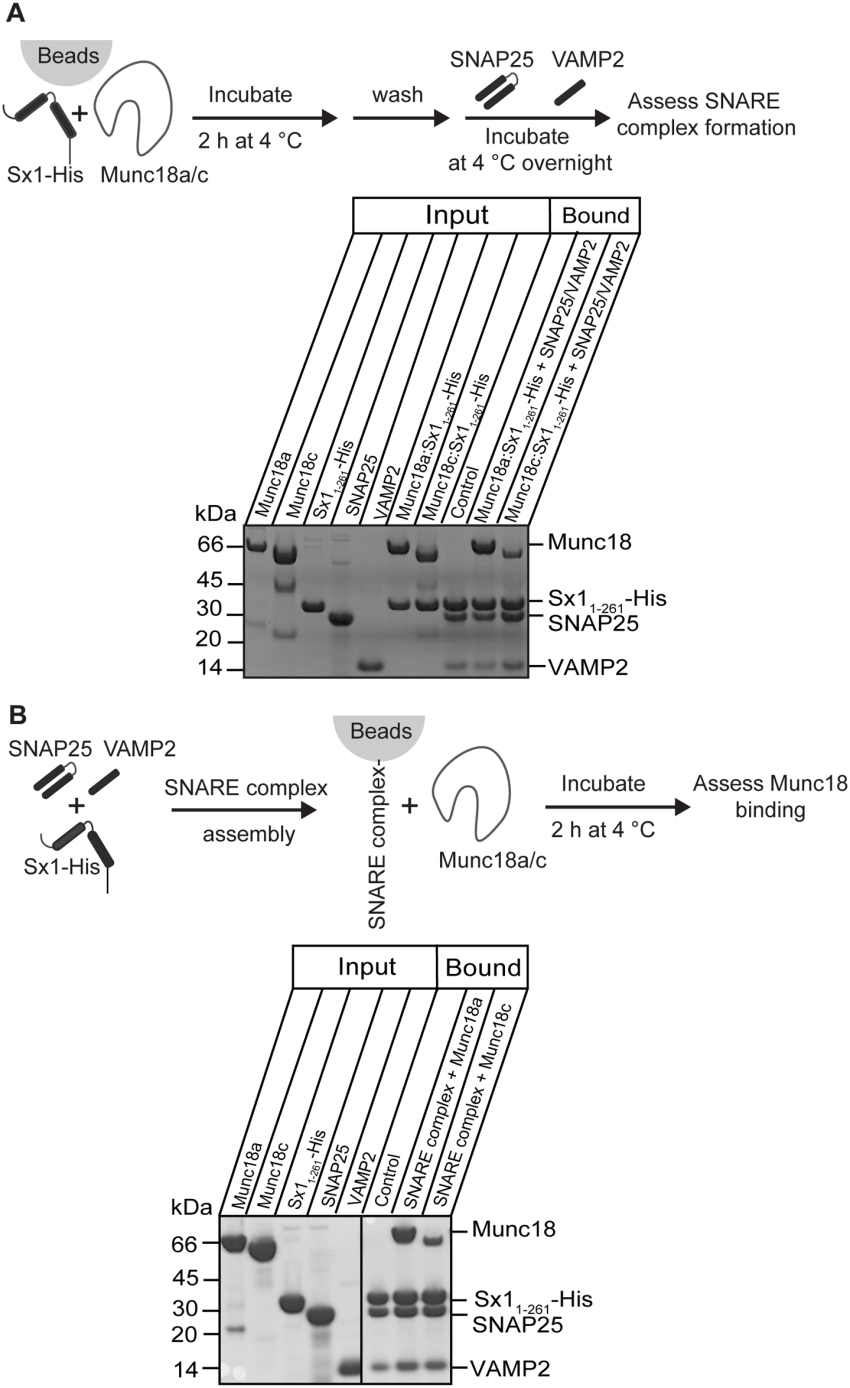
Non-cognate Munc18 interactions with SNARE complexes. (A) Cognate and non-cognate Munc18 proteins were first incubated with Sx1_1-261_-His to form the Munc18:Sx1_1-261_-His binary complex in the presence of beads. The beads were washed, then incubated with the Sx1 SNARE partners, SNAP25 and VAMP2, overnight at 4°C before SNARE complex formation was assessed by the presence of SNAP25 and VAMP2 on the beads (analysed by SDS-PAGE with Coomassie Blue staining). (B) Pre-formed SNARE complex comprising Sx1_1-261_-His:SNAP25:VAMP2 was captured by beads and then incubated with detagged Munc18a or detagged Munc18c for 2 h at 4°C. The SNARE complex was captured using affinity beads, and the presence of bound Munc18 was evaluated by SDS-PAGE analysis and Coomassie Blue staining. Each image shown is representative of multiple replicate experiments, and the solid lines through the gel indicate where images of different gels have been joined.

To determine whether non-cognate binary complexes were capable of binding SNARE partners *in vitro*, we used detagged Munc18c pre-formed in a binary complex with “non-cognate” Sx1. We chose this binary complex as it was the more stable of the two non-cognate complexes, enabling the use of stringent washing steps in the bead assays. In this experiment (Fig 5A) we demonstrated that Sx1_1-261_-His in complex with either detagged (cognate) Munc18a or detagged (non-cognate) Munc18c was able to capture SNARE partners SNAP25 and VAMP2. After the stringent washing steps there was a lower proportion of Munc18c present than at the beginning of the experiment, and less Munc18c than Munc18a, consistent with the relative binding affinities.

Munc18 proteins have also been shown to interact with pre-formed SNARE complexes [14, 23–26]. We tested whether non-cognate Munc18c could bind pre-formed neuronal SNARE complexes using pulldown experiments and found that pre-formed SNARE complexes containing Sx1_1-261_-His, SNAP25 and VAMP2 were able to capture Munc18c, though once again not as avidly as the SNARE complex captured Munc18a (Fig 5B).

## Discussion

SM:SNARE systems appear to have evolved to tailor their functions to specific isoforms. We observed that there is apparent redundancy in Munc18:Sx interactions, from the results of *in vitro* pulldown and ITC experiments. Overall, the results show that Munc18 proteins are able to bind non-cognate Sxs, though with varying levels of affinity. Specifically, Munc18a can bind non-cognate Sx4 weakly and Munc18c can bind non-cognate Sx1 tightly. However, the binding affinity of cognate Sxs varied tremendously: Munc18a bound Sx1 30,000 times more tightly than it bound Sx4, whereas—most surprisingly—Munc18c binds Sx1 a little more tightly than it binds Sx4. We note that the *in vitro* data were collected using Sx constructs lacking a C-terminal transmembrane domain. This means that the Sxs were not immobilised by their C-terminus in the ITC experiments—though all other experiments included a final step with the Sx C-terminus immobilised. We have shown previously that C-terminal immobilisation may impact on the outcomes of experiments and conclusions [27, 28]. Further, structural studies confirm the importance of the C-terminal region by showing that the C-terminal transmembrane domains of SNARE proteins interact with each other [29]. The impact of the transmembrane anchor and the plasma membranes on SNARE interactions with Munc18 proteins is not known, though both could play a role in binding mode and affinity.

**Fig 6:**
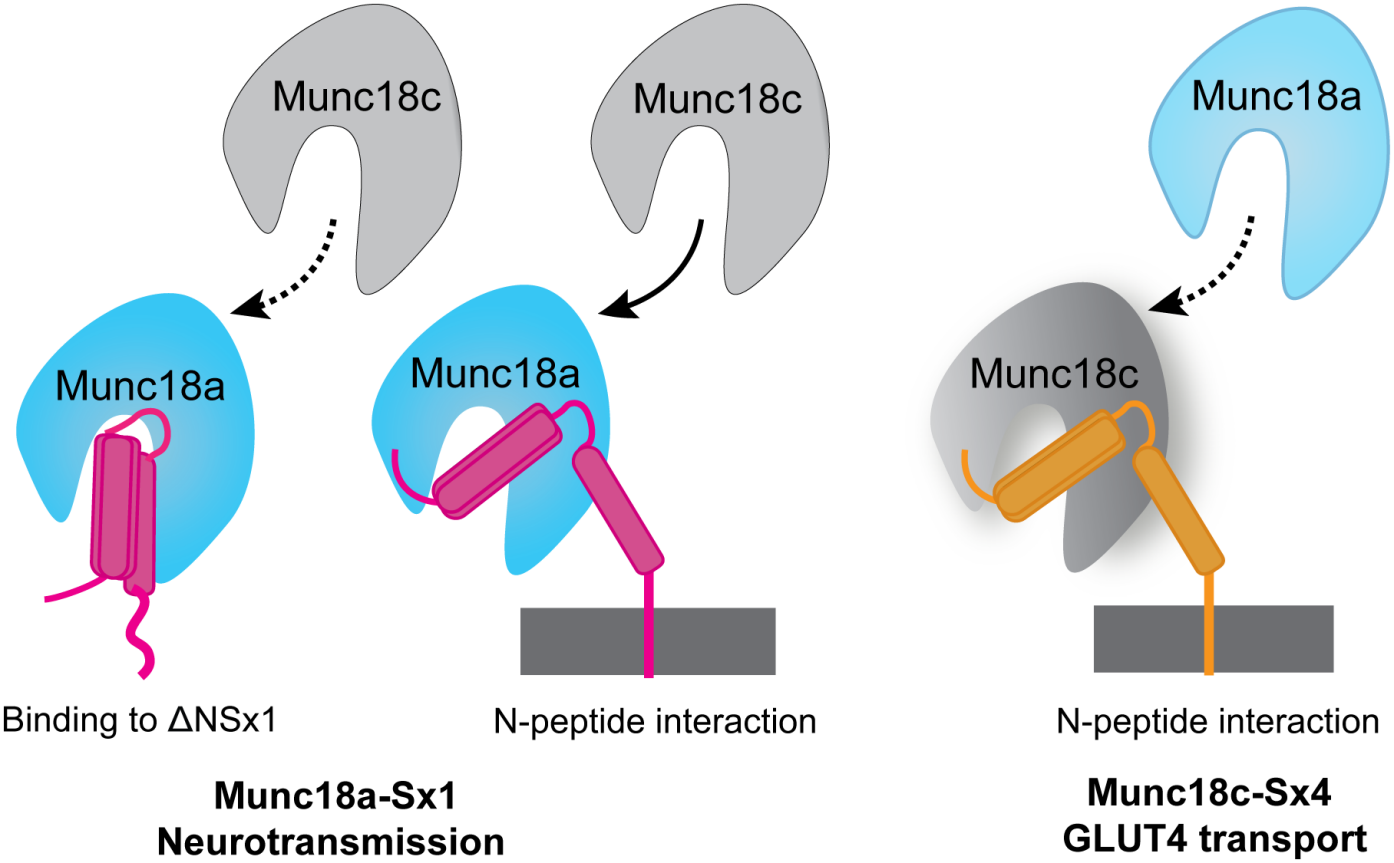
Specificity of Munc18:Syntaxin interactions. Munc18a (cyan) binds Sx1 (magenta) via two tight binding modes (left hand side). One binding mode occurs in the presence of the Sx1 N-peptide, the other in its absence. “Non-cognate” Munc18c (gray) also binds tightly to Sx1, though its interaction with Sx1 lacking the N-peptide is very weak (indicated by dotted line). Munc18c binds more tightly than Munc18a to Sx4 (orange) but neither Munc18 recognises Sx4 lacking its N-peptide. These findings indicate that Munc18a and Munc18c bind Sxs differently. Specifically Munc18a has two tight binding modes/sites for Sx1 one of which does not require the N-peptide binding interaction. Munc18c has one tight binding mode/site for Sx4 or Sx1 that requires the Sx N-peptide.

Nevertheless, an important finding was that in the absence of the N-peptide, the only tight binding complex of the four Munc18:ΔNSx pairs was that between the cognate Munc18a and ΔNSx1 proteins. ΔNSx4 had no detectable binding by ITC to Munc18c or Munc18a, and ΔNSx1 bound very weakly to Munc18c. This finding suggests that the N-peptide interaction is critical for Munc18c binding to Sxs, and for Sx4 binding to Munc18 proteins. This is not the case for Munc18a:ΔNSx1, and supports previous findings that Sx1 has at least two tight binding modes (one with, the other without, the Sx1 N-peptide) [19, 21, 22, 30, 31]. By contrast, the N-peptide binding mode contributes the majority of the binding interaction of Sx4 whether with “cognate” Munc18c or “non-cognate” Munc18a (Fig 6). The evidence presented here suggests that there is a second Sx binding site (in addition to the N-peptide site) on Munc18c, but that this second interaction is very weak. Overall, these results confirm that neuronal Munc18a:Sx1 has two tight binding modes, and show that Munc18 binding of Sx without its N-peptide is important for the neurotransmission Munc18:Sx interactions but this binding mode is not important for adipocyte GLUT4 transport Munc18:Sx interactions.

The binary Munc18:Sx interaction is likely an intermediate step in the vesicle fusion process. The interaction between Munc18 and Sx has been shown to be important for localizing Sx to the target membrane [32, 33] and in vesicle docking [34] and priming [35]. Munc18 proteins also bind to SNARE ternary complexes and can facilitate or block their formation [12–14, 22, 30, 36–41]. Our findings show that Munc18c can recognise the “non-cognate” Sx1 SNARE complex, indicating that this interaction (like the binary interaction with Sx1) may not fully explain the specificity observed for vesicle fusion.

Taken together, our data indicate that the current paradigm for Munc18 and Sx interaction specificity needs to be revised [13, 16–18]. Earlier work found no interaction between Munc18c and Sx1, but clearly the “non-cognate” Munc18c:Sx1 interaction is strong under the conditions we used. This difference in affinity compared with earlier work may be due in part to different experimental designs. For example Tellam *et al* [17] and Tamori *et al* [18] used N-terminally tagged Sx constructs to define Munc18:Sx specificity, and perhaps the tag impacted on the N-peptide interaction of Sx1 with Munc18c [17, 18]. At the time, the importance of the Sx N-peptide for Munc18c interactions was not known (and removal of the N-peptide from Sx1 makes only a 10-fold difference to its binding affinity for Munc18a).

The findings reported here suggest that the specificity observed in different Munc18:Sx fusion events may be affected by differential expression, by the existence of more than one binding mode or of different binding modes, and by binding thermodynamics.

In light of our new data, factors that we have not controlled for, such as the SNARE transmembrane anchors and the plasma membrane, may also contribute to Munc18:Sx specificity.

## Materials and methods

### Plasmid constructs

Constructs used in the experiments are shown schematically in Fig 1. Details are provided below.

#### Syntaxins

The cytoplasmic regions (lacking the transmembrane domain) of all the Sxs were expressed *in E*. *coli* with an engineered C-terminal 6x-His tag [21]. Rat Sx4 (residues 1–275, C141 replaced with Ser for ease of purification) was cloned into pET20b and rat Sx1 (residues 1–261) was cloned into pET24a. Sxs with N-terminal deletions, ΔNSx4 (residues 30–275, again with C141S) and ΔNSx1 (residues 25–261) were cloned into pET24a.

#### Munc18c

Two constructs of Munc18c were used: HMunc18c (N-terminal His_6_-tag) and Munc18c with an N-terminal His_6_-tag and a TEV cleavage site. Mouse Munc18c (residues 1–592) with a non-cleavable N-terminal 6x-His tag was generated using the pQE30 vector. This was modified to contain a TEV protease cleavage site to enable removal of the 6x-His tag, [42] and expressed in *E*. *coli*. The latter construct is referred to as detagged Munc18c in the manuscript.

#### Munc18a

Two constructs of Munc18a were used; both were expressed in *E*. *coli*. Rat Munc18a (residues 1–594) was cloned into a pGEX-KG vector to generate GST-Munc18a. The protein used in experiments described here was detagged Munc18a, with the tag removed using thrombin. Munc18a-His was generated by engineering a C-terminal 6xHis-tag using pET28a [19].

#### SNARE partners

Mouse SNAP25 (full-length, amino acids 1–206) was cloned into the ligation-independent cloning (LIC) vector pMCSG7 encoding an N-terminal polyhistidine tag with a tobacco etch virus (TEV) cleavage site [43]. VAMP2 lacking its transmembrane domain (amino acids 1–96) was engineered into pGEX-KG. Both SNAP25 and VAMP2 were expressed in *E*. *coli*. Detagged proteins (which we refer to as SNAP25 and VAMP2) were used for all experiments.

### Protein expression and purification

#### Syntaxins

All Sx constructs: Sx4_1-275_-His, Sx1_1-261_-His, ΔNSx4_30-275_-His, ΔNSx1_25-261_-His were expressed in BL21(DE3)pLysS cells in ZYP-5052 media by autoinduction [44]. Proteins were purified as described previously [19, 21]. Briefly, cells were lysed in 25 mM Tris-HCl (pH 7.5) buffer with 300 mM NaCl, 10% glycerol, 1% Triton X-100, 12,500–14,000 units DNase (Roche), 100 μL of protease inhibitor cocktail III (AG Scientific, Inc.), and 2 mM β-mercaptoethanol (β-ME). The lysate was cleared by centrifugation and applied to Ni^2+^ chelated PrepEase^™^ resin (USB Corporation) or TALON^™^ Co^2+^ affinity resin (Clontech) for metal affinity purification. Bound protein was washed in: 25 mM Tris-HCl (pH 7.5), 300 mM NaCl (wash buffer) wash buffer with 10 mM imidazole (100 mL) then wash buffer with 20 mM imidazole (50 mL). Bound Sx was eluted in wash buffer containing 300 mM imidazole. Proteins were further purified by anion exchange chromatography on a MonoQ 5/5 column (GE Healthcare, UK) [19].

#### Munc18c

HMunc18c in pQE30 vector was co-transformed into *E*. *coli* BL21 cells along with pREP4 plasmid encoding GroEL/ES chaperones [42]. HMunc18c was expressed in autoinduction media [44] at 37°C until an OD_600_ of 0.5–0.6 was reached, expression was then continued at 16°C overnight. HMunc18c was purified by resuspending BL21 cells in 25 mM Tris-HCl pH 7.5 buffer containing 300 mM NaCl, 10% (v/v) glycerol, 10 mM imidazole, 2 mM βME, 1% (v/v) Triton X-100, 0.5 mM EDTA, 100 μL of Bacterial Protease Inhibitor (BioPioneer, Inc., USA). They were then homogenized by passing through a disposable syringe and lysed by addition of lysozyme (400 μg/mL, Astral Scientific, Australia) followed by incubation at 4°C for 1 h. After addition of 13,000 U of DNase the sample was incubated for a further hour at 4°C with regular mixing by syringe. When the solution became less viscous (free-flowing) the cell lysate was cleared by centrifugation. The cleared lysate was applied to PrepEase^™^ Ni^2+^ chelated resin (USB Corporation, USA) and incubated at 4°C for 2 h. The resin was washed with wash buffer (25 mM Tris-HCl pH 7.5, 300 mM NaCl, 10% (v/v) glycerol, 2 mM β-ME) containing 10 mM imidazole followed by wash buffer with 25 mM imidazole, to remove contaminants. HMunc18c was eluted with 300 mM imidazole in wash buffer. The protein in the eluant was further purified by size exclusion chromatography (SEC) on a Superdex 200 16/60 (S200) using an ÄKTA FLPC system (GE Healthcare) in SEC buffer (25 mM HEPES pH 8.0, 200 mM NaCl, 2 mM β-ME, 10% (v/v) glycerol buffer) [42] followed by cation exchange chromatography using SEC buffer and a 0.2 M to 1 M NaCl gradient on a MonoS 5/5 column, if further purity was required.

Detagged Munc18c was expressed and purified as described above up to elution from PrepEase^™^ Ni^2+^ chelated resin. Munc18c eluted from the resin was then mixed with TEV protease (10:1 protease to protein ratio) and dialysed overnight at 4°C into 25 mM HEPES pH 8.0, 200 mM NaCl, 2 mM β-ME, 10% (v/v) glycerol buffer with 10 mM imidazole. Detagged Munc18c was separated from uncut Munc18c and TEV protease and further purified by metal affinity chromatography using PrepEase^™^ Ni^2+^ chelated resin followed by SEC in 25 mM HEPES pH 8.0, 200 mM NaCl, 2 mM β-ME, 10% (v/v) glycerol buffer.

#### Munc18a

Munc18a constructs (either in pGEX-KG or pET28a) were expressed and purified essentially as described in Hu et al. [19]. Both constructs were expressed in *E*. *coli* BL21(DE3)pLysS cells by autoinduction [44].

His-tagged Munc18a was lysed in 50 mM phosphate buffer pH 8.0 containing 500 mM NaCl, 10% glycerol, 1% Triton X-100, 12,500–14,000 units DNase (Roche), 100 μL of protease inhibitor cocktail III (AG Scientific, Inc.), and 2 mM β-ME. Cleared lysate was applied to PrepEase^™^ resin (USB Corporation) and incubated for 30 min before washing with wash buffer containing 50 mM phosphate buffer pH 8.0 containing 500 mM NaCl, 10% glycerol and 2 mM β-ME with first 10 mM and then 20 mM imidazole. Bound Munc18a was eluted with wash buffer that included 300 mM imidazole and purified by size exclusion chromatography on a Superdex 200 16/60 (S200) (GE Healthcare, UK) in 25 mM HEPES pH 8.0, 200 mM NaCl, 2 mM β-ME, 10% (v/v) glycerol buffer [42].

GST-cleaved Munc18a was prepared by lysing cells in 25 mM HEPES, pH 7.4, 500 mM NaCl, 5 mM DTT and 0.5% Triton X-100 with 1× Complete EDTA-free protease inhibitor cocktail tablet (Roche), 1 mM EDTA and 8,000–10,000 units DNase I. GST-Munc18a was bound to glutathione agarose resin by incubating the cleared lysate with resin for 2 h at 4°C. After washing in 25 mM HEPES, pH 7.4, 500 mM NaCl, 10% glycerol and 5 mM DTT, Munc18a was cleaved by treatment with thrombin in 25 mM Tris pH 8.0, 300 mM NaCl, 5 mM DTT, 10% glycerol, 6 mM CaCl_2_ buffer. Thrombin was inactivated by addition of protease inhibitors (1 mM AEBSF, 1× Complete EDTA-free protease inhibitor cocktail tablet) and 1 mM EDTA. Thrombin was removed using ion exchange chromatography on a MonoQ 5∕5 anion exchange column [19] (GE Healthcare Biosciences).

#### SNARE partners

His-TEV SNAP25 was produced and purified as described previously [19]. The protein was lysed in lysis buffer containing 25 mM Tris-HCl, pH7.5, 300 mM NaCl, 0.5% Triton-X 100, 12,500–14,000 units DNase (Roche), 100 μL of protease inhibitor cocktail III (AG Scientific, Inc.) and 2 mM β-ME. The cleared lysate was purified using PrepEase Ni^2+^-chelated resin (USB Corporation). To remove the His tag, purified His-tagged SNAP25 was incubated at 4°C overnight with His-tagged TEV protease. The TEV protease was removed with Co^2^-affinity beads (Clontech), and the detagged SNAP25 was further purified using size exclusion chromatography (Superdex 75 16/60) (GE Healthcare, UK) [19].

GST-cleaved VAMP2 was produced and purified as described previously [12]. The protein was lysed in lysis buffer containing 25 mM Tris-HCl, pH7.5, 300 mM NaCl, 0.5% Triton-X 100, 12,500–14,000 units DNase (Roche), 100 μL of protease inhibitor cocktail III (AG Scientific, Inc.) and 2 mM β-ME. The cell lysate was centrifuged using JA25.5 rotor, AVANTI centrifuge (Beckman Coulter, USA) at 18,500 rpm, 30 min at 4°C and the cleared supernatant was incubated with GSH-agarose resin (Thermo-Fisher Scientific, Massachusetts, USA) for 2 hrs. The beads were then washed with wash buffer (25 mM Tris-HCl, pH7.5, 150 mM NaCl, 2 mM β-ME) prior to treatment with thrombin (10 U/ml in solution) to cleave the GST affinity tag. Proteolysis was stopped by addition of the protease inhibitor AEBSF (1 mM at final concentration). The cleaved VAMP2 was further purified by cation exchange chromatography on a MonoS HR 5/5 column (GE Healthcare, UK).

### Binding experiments

The purified His-tagged Syntaxin proteins (Sx4_1-275_-His, Sx1_1-261_-His -His, ΔNSx4_30-275_-His and ΔNSx1_25-261_-His) were incubated with detagged Munc18 proteins for 2 h, 24 h and 48 h at 4°C (1:2 molar ratio). They were then incubated with TALON^™^ Co^2+^ affinity resin (Clontech) for 1h at 4°C. The beads were washed with binding buffer (25 mM TrisHCl pH 7.5, 150 mM NaCl, 10% glycerol, 0.1% Triton-X 100, 15 mM imidazole, 2 mM β-ME) to remove any excess protein and analysed using SDS-PAGE.

Pulldown experiments to determine if Munc18c and non-cognate Sx complex enabled SNARE complex formation were carried out as follows. Detagged Munc18c was incubated with His-tagged cognate and non-cognate Sx and TALON^™^ Co^2+^ affinity resin for 2 h at 4°C. Samples were then washed with binding buffer to remove any excess unbound Munc18c. Washed beads were incubated overnight at 4°C with SNAP25 and VAMP2 before washing off any unbound proteins. SDS-PAGE analysis was then carried out to determine complex assembly. (Similar experiments on detagged Munc18a were not under taken as Munc18a and non-cognate Sx4_1-275_-His did not form 1:1 complex even after 48 h incubation on beads.)

The gels shown are representative of at least three replicate experiments for each binding experiment.

### Isothermal titration calorimetry

ITC experiments were carried out between Munc18 proteins (HMunc18c or Munc18a-His) at 10–20 μM concentrations with both cognate and non-cognate Syntaxin partners (100–550 μM). All experiments were carried out at 298 K in 25 mM HEPES pH 8, 200 mM NaCl, 2 mM β-mercaptoethanol and 10% glycerol. 16 injections of 2.45 μL were used for each experiment. The heat released was integrated using Microcal ORIGIN 7 software to yield the stoichiometry (*N*), equilibrium constant *K*_a_ (1/*K*_d_) and binding enthalpy of interactions (Δ*H*). The Gibbs free energy (Δ*G*) was calculated using the equation: *ΔG* = −*RTln*(*K*_*a*_), binding entropy (Δ*S*) was calculated using *ΔG* = *ΔH* − *TΔS*. The reported values are the average and standard deviation of results of at least three experiments.
